# Lipid profiles of female and male *Drosophila*

**DOI:** 10.1186/1756-0500-4-198

**Published:** 2011-06-15

**Authors:** Michael Parisi, Renhua Li, Brian Oliver

**Affiliations:** 1 Department of Biology, University of Pennsylvania, 415 South University Avenue, Philadelphia, PA 19104, USA; 2 Section of Developmental Genomics, Laboratory of Cellular and Developmental Biology, National Institute of Diabetes and Digestive and Kidney Diseases, National Institutes of Health, Department of Health and Human Services, 50 South Drive, Bethesda MD 20892, USA

## Abstract

**Background:**

*D. melanogaster *is increasingly used as a lipid metabolism model, but the *D. melanogaster *metabolome is not well studied. A number of studies strongly suggest that lipid metabolism is linked to sexual behavior and gametogenesis.

**Findings:**

We determined the levels of 400 different lipids in the non-gonadal soma of *D. melanogaster *females and males. We found higher levels of saturated cholesterol esters and lysophosphatidylcholine in males, and higher levels of polyunsaturated cholesterol esters in females. We also determined the levels of these lipids in females and males without a germline to determine if the absence of gamete "sinks" for metabolic products, such as yolk and lipid deposits in eggs, altered somatic lipid profiles. We observed little change in lipid profiles between these samples.

**Conclusions:**

Overall lipid compositions are similar between the sexes, although there are differences in saturation states of two lipid classes, where saturated fatty acids were male-biased and polyunsaturated fatty acids were female-biased. The presence of a germline did not significantly influence lipid profiles, raising the possibility that germline-dependent changes in metabolic gene expression patterns serve a homeostatic purpose.

## Background

Lipids are the major energy storage molecules in cells and act as ligands in cell-cell and organism-organism pheromone signaling. *Drosophila *is an emerging model for studying all of these aspects of lipid biology [1-4]. We are particularly interested in sex differentiation and there is much indirect evidence that energy storage, cell-cell signaling, and pheromone lipid requirements differ between the *Drosophila *sexes.

The energy storage needs of females are higher than those of males due to egg production. Eggs, which are comprised primarily of lipoprotein particles (yolk) to store energy for embryonic development, make up a large fraction of the female's body mass and are therefore a metabolically expensive energy sink [5,6]. The lipid signaling molecule ecdysone, best known for the role it plays in metamorphosis [7], is highly female-biased in adults [8] and plays a major role in production of yolk constituents in the ovarian somatic follicle cells and distantly located fatbody where they are transported to growing oocytes via the hemolymph [5,9-11]. Metabolic enzymes such as the digestive chymotrypsins also show sex-biased expression in *Drosophila *[8,12], again supporting the idea of a link between reproduction and energy homeostasis.

In addition to the direct connections between egg and lipid production, a number of lipids act as sex-biased hormones or pheromones that modulate pre- and post-mating behaviors in flies [13,14]. These lipids might play a regulatory role in linking energy storage and reproduction. For example, the head fatbody shows sex-biased and/or circadian expression of a host of genes that encode lipid-binding proteins, some of which regulate feeding behavior, mating, or both [15-19]. Interestingly, the gene encoding the critical transcriptional regulator of most aspects of somatic sex differentiation, Doublesex, is expressed in a tightly regulated and spatially restricted set of cells in the nervous system, the fatbody, and a segment of the midgut where it is well positioned to modulate lipid metabolism in the full spectrum of cell types that might regulate a physiological axis including the brain, fatbody, and digestive tract of the sexes [20,21]. Fruitless, another transcription factor controlling mating behavior is expressed in a limited set of neurons in *Drosophila *[22], and also regulates lipid storage [23]. These studies suggest that the sex determination hierarchy is a regulator of energy homeostasis.

Such physiological relationships are perhaps best observed in fitness trade-off experiments that explore the competing optimal conditions for somatic and germline development. For example, reproduction reduces the lifespan of *C. elegans *and alters lipid metabolism [24,25]. In *Drosophila*, increased egg production results in starvation sensitivity, and conversely, blocking egg maturation prevents a metabolic shift in the acid/base balance in the female gut at the onset of young adult female reproductive activity [26,27]. These and other studies suggest that lifespan, reproduction, and energy metabolism are linked in both *Drosophila *and *C. elegans *[2]. We have previously reported germline-dependent changes in the expression of genes encoding metabolic functions and suggested that they may underlie some of these metabolic/reproductive phenotypes [8]. To support future work on lipid metabolism as it relates to sex, we undertook a broad survey of lipid profiles in adult non-gonadal tissues. We also explored the possible influence of the germline on these profiles.

## Findings

To obtain a reasonably comprehensive profile of lipids in the Drosophila soma, we examined 10 lipid classes: cardiolipin, cholesterol ester, diacylglycerol, free fatty acid, lysophosphatidylcholine, phosphatidylcholine, phosphatidylethanolamine, phosphatidylserine, sphingomyelin, and triacylglycerol by mass spectrometry (Lipomics Technologies, Sacramento CA). We made lipid determinations on mated sexed adult flies of the genotype *tud^1 ^bw^1 ^sp^1^/CyO *at 5-7 days after eclosion. To eliminate direct germline contributions to the lipid profiles, we removed the gonads prior to extraction. This also results in the loss of hemolymph and therefore most of the circulating lipids. To determine if lipid profiles differed due to indirect effects of the germline on somatic physiology, we examined flies from homozgyous *tud^1 ^*mothers. The progeny of homozygous *tud^1 ^*mothers do not form a germline, while progeny of heterozygous *tud^1 ^*mothers have a fully functional germline. This allowed us to examine the effect of the germline on somas with the same zygotic genotype. This is one of the same maternal/zygotic genotypes we previously described for expression profiling [8]. Flies were grown on a standard rich cornmeal/sugar/yeast/agar media (<https://stockcenter.ucsd.edu/info/food_cornmeal.php>, Drosophila Species Stock Center, Tucson AZ); at 22°C; with 60% relative humidity; under constant light. We obtained lipid profiles from 8 samples, 4 from each sex, further stratified by germline status (Additional File 1). Note that statistical power was strongest for overall lipid profiles in adult flies where sample size was 8 and weakest for germline status within sex where sample size was 2. Because of the limited differences in lipid levels observed, collapsing germline classes to increase power was statistically justified by homogeneity.

**Figure 1:**
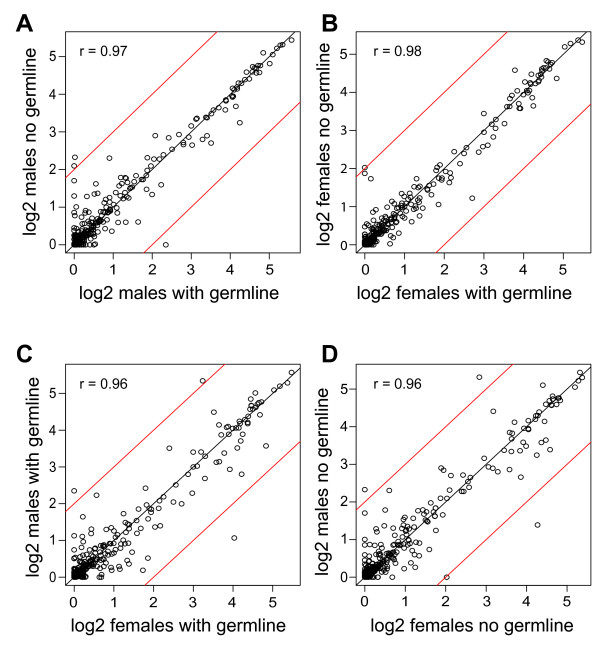
**Relationships of fatty acids between germline states and between sexes**. (A, B) Within sex comparisons between flies with a germline or with no germline. (C, D) between sex comparisons of flies with a germline or with no germline. Each data point represents the between-replicate mean value (on a log2(x+1) scale; where x is the measured value) of a fatty acid. The red lines indicate the limits of 95% confidence intervals, based on bootstrap resampling methods [33].

To compare all of the fatty acids profiled, we plotted the data for individual lipid species within the ten major classes by germline state and by sex. We found remarkably little difference in lipids between flies with or without a germline (r > 0.97; Figure 1a,b) and only slightly increased scatter between the sexes (r = 0.96; Figure 1c,d). We did observe a few data points outside the 95% confidence interval limits, but outliers are expected among the 400 lipids measured. However, this exploratory analysis showed that all the outliers are members of the sphingomyelin, cholesterol ester, and lysophosphatidylcholine classes, suggesting that the outliers represent more than random measurement error. Our suspicions were raised further by the pattern of saturation states among the outliers. The species higher in males were saturated 16 or 18 carbon fatty acids (16:0 or 18:0) and the species higher in females were monounsaturated 18 carbon fatty acids (18:1n7) and polyunsaturated fatty acids (18:3n6, 20:3n3, or 20:3n9). These data suggested that saturation status within a lipid class might be sex-biased.

**Figure 2:**
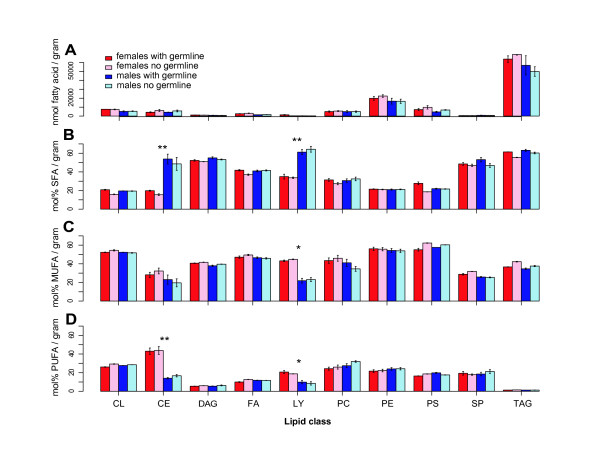
**Distribution of fatty acids in the sexes with a germline or with no germline**. (A) Fatty acids in nmoles per gram tissue by lipid class. Varied fatty acid components of lipids can be grouped into three categories: (B) saturated fatty acids (SFA); (C) monounsaturated fatty acids (with one double bonded carbon; MUFA); and (D) polyunsaturated fatty acids (with multiple double bonded carbons; PUFA). Female and male nongonadal somas from flies with a germline or with no germline were assayed (see key). Lipids were grouped into ten lipid classes: CL = Cardiolipin; CE = Cholesterol ester; DAG = Diacylglycerol; FA = Free fatty acid; LY = Lysophosphatidylcholine; PC = Phosphatidylcholine; PE = Phosphatidylethanolamine; PS = Phosphatidylserine; SP = Sphingomyelin; TAG = Triacylglycerol. Histograms are mean ± SEM. Significant differences between sexes after binning the with and with no germline data (* p < 0.05; ** p < 0.005, *t-*test).

To test this hypothesis, we grouped the lipids by class. By abundance, the storage molecule triacylglycerol was the dominant class of lipid in the adult soma, followed by the lipid bilayer components lysophosphatidylcholine, phosphatidylcholine, and phosphatidylethanolamine. As suggested by plotting the abundances of the individual lipids, there were no significant differences in the abundances of the 10 major classes in non-gonadal soma between the sexes, or in flies with or without a germline (Figure 2a).

We then binned lipid classes into saturated, monounsaturated, and polyunsaturated fatty acids. Again, we observed no significant differences between the flies with or without a germline within each sex, but we did observe sex-bias in the saturation states of cholesterol esters and lysophosphatidylcholines (Figure 2b-d). Since we observed no significant differences due to germline status (p > 0.05, *t*-test), we treated these within-sex samples as an additional level of replication in order to increase the power of statistical tests for the differences in lipid saturation between sexes. As suggested by the initial exploratory analysis, we observed significantly higher saturated cholesterol ester and lysophosphatidylcholine levels in males (p < 0.005, *t*-test) and an increase in polyunsaturated and/or monounsaturated cholesterol ester and lysophosphatidylcholine levels in females (p < 0.05, *t-*test). Given that lecithin:cholesterol acyltransferase transfers fatty acids from phosphatidylcholine to form cholesterol ester and lysophosphatidylcholine, these differences in saturation states may be linked.

**Figure 3:**
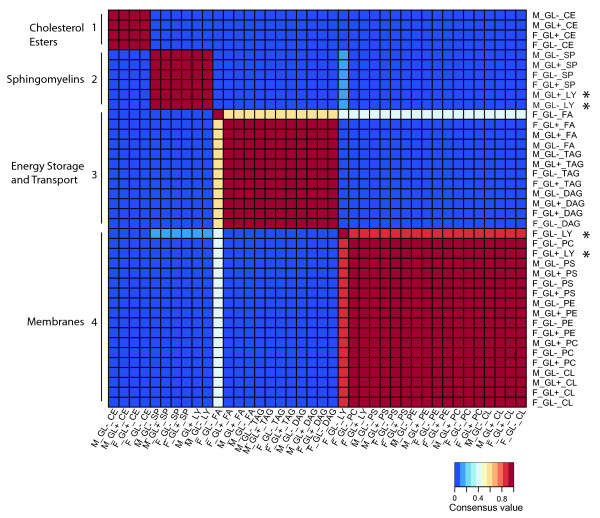
**A heatmap of sample-lipid class combinations**. Given the matrix partition factor k = 4, we ran the NMF algorithm 100 times to generate consensus clustering of the combinations of samples and lipid classes. Each run resulted in a 40 × 40 connectivity matrix with an entry of 1 if sample-lipid class combinations i and j cluster together and 0 otherwise, where i, j = 1,..., 40. The consensus matrix is the averaged connectivity matrix obtained over the 100 runs. Each block along the main diagonal represents a consensus cluster of the combination of samples and lipid classes. Meta-fatty acid cluster characteristics are to the left, specific meta-fatty acid groups to the right: F = female; M = male; GL+ = with a germline; GL- = with no germline; CL = cardiolipin; CE = cholesterol ester; DAG = diacylglycerol; FA = free fatty acid; LY = lysophosphatidylcholine; PC = phosphatidylcholine; PE = phosphatidylethanolamine; PS = phosphatidylserine; SP = sphingomyelin; TAG = triacylglycerol. Only Lysophosphatidylcholine from males and females map to different locations in the matrix (*).

We were interested in further examining the relationships between the different lipids to determine if particular lipids co-vary, or cluster, among the samples. This type of analysis is particularly useful with limited sample sizes as there are many more measurable relationships between lipid species than between samples. We used nonnegative matrix factorization (NMF), an unsupervised, parts-based learning paradigm, to explore these relationships [28]. The fatty acid profiling data was input as a matrix with cells representing the 40 combinations of samples and lipid classes that is decomposed into weight and pattern via a multiplicative updates algorithm [28] to estimate that there were 4 meta-fatty acid clusters (not shown). On the basis of k = 4, we generated four consensus clusters of the 40 combinations of samples and lipid classes (Figure 3). These distinct clusters are associated with specific biochemical functions. We observed only a single lipid class that mapped to different positions in the matrix as a result of sex. In males, lysophosphatidylcholine clustered with the sphingomyelins, while in females lysophosphatidylcholine clustered with the other membrane lipids. These data provide additional evidence that there is sexual dimorphism for this specific lipid class. However, the most striking finding is that the relationships between lipids are quite similar between the sexes and between flies with or without a germline.

## Discussion

Our *a priori *hypothesis was that lipid profiles would differ dramatically between sexes and especially between flies with or without a germline. We provide no evidence to support the hypothesis that lipid profiles in the non-gonadal soma are germline-dependent. However, we did observe sex-biased saturation states. It is intriguing that the saturation differences we observed were in the lysophosphatidylcholine and cholesterol ester classes, as lysophosphatidylcholine and cholesterol ester are produced by LCAT, an enzyme implicated in Low and High Density Lipoprotein particle formation [29]. *Drosophila *egg development relies on Low Density Lipoprotein particles that are taken-up from the hemolymph [5], which is also intriguing. But in the absence of eggs, we would have expected some change in the lysophosphatidylcholine or cholesterol ester profiles in the female soma. Thus the germline-dependent expression of genes encoding various lipid metabolism enzymes [8,27] is not mirrored by germline-dependent lipid profiles. One hypothesis is that those changes in gene expression maintain lipid homeostasis in the absence of a germline "sink" for lipids.

Saturation states have been implicated in mating behavior in flies. The *sex-specific enzyme 1 (sxe1*) locus encodes a putative fatty acid hydrolase required for high mating efficiency. In the absence of *sxe1 *the saturation states of multiple lipids are altered in male heads suggesting that lipid saturation plays a role in mating behavior [15]. The *lipid desaturase 1 *locus (*dsat1*) is required for both pheromone signaling and the starvation response in flies [30-32]. Our work suggests that the major lipid differences between the sexes are restricted to saturation states. Saturation states may be an area of further investigation for those interesting in tying together the emerging physiological axis that coordinates mating and feeding behavior with energy storage and gametogenesis.

## Acknowledgements

This research was supported by the Intramural Research Program of the NIH, National Institute of Diabetes and Digestive and Kidney Diseases.

## Competing interests

The authors declare that they have no competing interests.

## Authors' contributions

MP and BO conceived the project. MP performed all wet-bench work. RL performed all statistical analysis. MP, RL and BO analyzed data and wrote the manuscript.

## Supplementary Material

Additional File 1 **Lipid profiling data set**. Click here for file
